# A brief mindfulness intervention improves electrophysiological markers of attention in meditation-naïve individuals: the moderating role of inattention symptoms

**DOI:** 10.3389/fpsyg.2025.1629282

**Published:** 2025-08-21

**Authors:** Xiaoqian Yu, Christine Vinci, Geoffrey F. Potts

**Affiliations:** ^1^Department of Psychology, University of South Florida, Tampa, Fl, United States; ^2^School of Psychology, Wenzhou-Kean University, Wenzhou, Zhejiang, China; ^3^Department of Health Outcomes and Behavior, Mott Cancer Center, Tampa, FL, United States

**Keywords:** mindfulness, attention, inattention, ADHD, P3b, P300, oddball task, EEG

## Abstract

**Background:**

Attention regulation is crucial for mindfulness practice; however, the influence of baseline attention ability on mindfulness training outcomes remains underexplored. This study examined the effects of a brief mindfulness intervention on attention and investigated whether baseline inattention symptoms moderated these effects in meditation-naïve university students.

**Methods:**

This study employed a pretest-posttest, between-groups experimental design. Meditation-naïve university students (*n* = 121, aged 18–31, 69% women) were randomly assigned to either a mindfulness group, which engaged in 10 min of guided mindful breathing, or a control group, which listened to a talk on green living. Baseline attention was assessed using the Adult ADHD Self-Report Scale (ASRS), and attentional changes were measured via EEG recorded during a visual novelty oddball task.

**Results:**

Both groups showed increased post-intervention P3b amplitudes, an electrophysiological indicator of attention. However, the mindfulness group exhibited a significantly greater increase compared to the control group. Importantly, inattention symptoms moderated this effect: participants with higher inattention symptoms in the mindfulness group showed a greater increase in P3b amplitude, while those in the control group showed a decrease.

**Conclusion:**

These findings highlight the importance of considering individual attentional profiles when designing mindfulness-based interventions. Tailoring mindfulness training based on baseline attention levels may enhance its cognitive benefits. Future research should explore additional potential moderators of mindfulness training outcomes and clinical conditions (e.g., anxiety or depression) that may influence attentional functioning and responsiveness to mindfulness practice.

## 1 Introduction

In recent years, mindfulness training has emerged as a promising cognitive training tool with potential benefits for enhancing attentional control, emotional regulation, and mental health (Bian and Jiang, 2025; Guo et al., 2019, 2020; Lomas et al., 2018). Mindfulness training refers to the practice of “paying attention in a particular way: on purpose, in the present moment, and nonjudgmentally” (Kabat-Zinn, 1994). While numerous studies have investigated the benefits of extended mindfulness programs, a growing body of studies suggests that even brief sessions, lasting as little as 10 min, may yield measurable cognitive benefits, particularly in attention-related domains (Creswell, 2017; Norris et al., 2018). Recent research has advocated for an individual difference perspective in mindfulness training research (Tang and Braver, 2020). The degree to which individuals benefit from such brief interventions may depend on individual differences in baseline attentional capacity, including the presence of attention-deficit/hyperactivity disorder (ADHD) symptoms, even among healthy populations.

Although numerous studies have examined the impact of mindfulness on attention (Semple, 2010; Sumantry and Stewart, 2021; Van der Lubbe et al., 2018), few studies have considered individual differences in baseline attention capacity that might affect the training outcome of mindfulness. Research on the effects of individual differences on mindfulness training outcomes has revealed variations in effectiveness based on personal attributes such as personality traits (de Vibe et al., 2015; Nyklíček and Irrmischer, 2017), baseline levels of dispositional mindfulness (Greeson et al., 2015), and distress tolerance (Gawrysiak et al., 2016). Cumulative theoretical accounts of mindfulness have emphasized the central role of attention in mindfulness practice, including the two-component operational definition (Bishop et al., 2004), the three components (axioms) of mindfulness (Shapiro et al., 2006), and the Monitor and Acceptance Theory (Lindsay and Creswell, 2017). High baseline attentional control is believed to support mindfulness practice by reducing the effort needed to maintain present-moment focus, limiting mind-wandering, and sustaining awareness. Consequently, individuals with greater attentional control may achieve a more stable mindfulness state and receive greater training benefits.

ADHD is a prevalent neurodevelopmental disorder that is characterized by elevated levels of inattention and impulsivity persisting into adolescence and adulthood in about two-thirds of individuals (Ebejer et al., 2012). Notably, many individuals without a clinical diagnosis still show subthreshold symptoms that may impact daily functioning (Canu and Eddy, 2015; Kessler et al., 2005). In college students, up to 10.3% of individuals without a clinical diagnosis of ADHD exhibited high levels of ADHD symptoms (Garnier-Dykstra et al., 2010). College students who face high cognitive demands and stress and elevated levels of inattention and impulsivity have been linked to impaired academic performance, reduced interpersonal skills, and lower psychological resilience (Kwon et al., 2018; Sedgwick, 2018). Given the substantial variability in ADHD symptoms among healthy adults, it is plausible that inattention might modulate the efficacy of mindfulness-based interventions on attention enhancement.

Mindfulness training has been proposed to enhance attentional control and reduce the core symptoms of ADHD (i.e., inattention and impulsivity; Cairncross and Miller, 2020; Chimiklis et al., 2018). Emerging research suggests that mindfulness can enhance attentional performance in both clinical and non-clinical populations (Chiesa et al., 2011). One study reported that meditation-naïve undergraduate students showed improved executive attention after mindfulness practice. Specifically, compared to those who listened to a control tape, participants who listened to a 10-min meditation tape had better accuracy on incongruent trials on a Flanker task, faster response times on the attention network test, and larger N2 to incongruent trials, especially for individuals low in neuroticism (Norris et al., 2018). However, less is known about how individual differences in attention-deficit-related symptoms might influence the effectiveness of brief mindfulness exercises in adults.

The specific role of impulsivity in modulating mindfulness outcomes is less well understood. While some propose that structured mindfulness-based interventions such as mindfulness-based cognitive therapy (MBCT) may help reduce impulsive responding over time (Hepark et al., 2019), others reported that an 8-week mindfulness intervention did not reduce impulsivity as measured by either the Behavioral Inhibition System (BIS)-11 or the go/no-go task and did not result in any neurobiological changes compared to either active or wait-list control groups (Korponay et al., 2019).

The present study addresses a critical gap in the literature by investigating how baseline attention abilities and subclinical ADHD symptoms, particularly inattention, moderate the effects of mindfulness training on attention performance in college students. Using a pre-post, between-groups experimental design, participants completed a 3-stimulus novelty oddball task while having their EEG recorded. We predicted that compared to the control group, the mindfulness group (1) would show a larger P3a to novel stimuli and a larger P3b to target stimuli in the post-intervention novelty oddball task; (2) would respond faster to target stimuli in the post-intervention novelty oddball task; and (3) the above effects would be more pronounced in individuals low in ADHD symptoms, as they might find it easier to comply with mindfulness practice due to higher baseline attention ability. The findings have significant implications for tailoring mindfulness-based interventions in educational and clinical settings, as well as for understanding individual differences in cognitive malleability. By identifying factors that predict differential responsiveness to brief mindfulness training, this study aims to contribute to the development of more personalized and effective attention-enhancement strategies for college students.

## 2 Materials and methods

### 2.1 Participants

This study employed a pre-post experimental design with a between-groups structure, comprising a Mindfulness group and a Control group. An a priori power analysis was conducted using G^*^Power 3.1 (Faul et al., 2009) to determine the required sample size for a repeated measures ANOVA with a between-subjects factor. The analysis specified a medium effect size (*f* = 0.25), an alpha level of 0.05, and a desired power of 0.85. The design consisted of two groups, mindfulness and control, and two measurement time points: pre- and post-intervention. Based on these parameters, a total sample size of ~60 participants was recommended (30 per group) to adequately detect the anticipated effects. To account for potential attrition and EEG data quality, 121 undergraduate participants were recruited from the Department of Psychology at the University of South Florida. A pre-screen survey was administered to determine their eligibility. Participants were eligible if they were between 18 and 31 years old, English-speaking, with intact hearing and normal or corrected-to-normal vision. Event-related potentials (ERPs) can be influenced by handedness. Previous studies have shown that compared to right-handers, left-handers exhibited larger P300 amplitudes and shorter latencies across auditory and visual oddball tasks (Hoffman and Polich, 1999; Polich and Hoffman, 1998). Therefore, to reduce variability in brain lateralization and ensure more consistent neural activation patterns, only right-handed participants were included in the study. Exclusion criteria were: 1) prior experience with any kind of practice featuring mindfulness (e.g., meditative yoga, Tai Chi, Qigong); 2) the presence of substance abuse (i.e., alcohol, nicotine, or illicit drugs); and 3) a history of neurological disorders per self-report. Participants earned four course credits for participating in the study. The study protocol was approved by the Institutional Review Board at the University of South Florida.

### 2.2 Measures and equipment

All surveys were administered in English through Qualtrics (Qualtrics, Provo, UT). Demographic information, including gender, age, ethnicity, and race, was recorded.

#### 2.2.1 Mindful attention awareness scale (MAAS)

The 15-item MAAS was designed to assess trait mindfulness: attentiveness and awareness (Brown and Ryan, 2003). The MAAS was administered before the study to examine pre-existing group differences in dispositional mindfulness. A sample item is, “I find it difficult to stay focused on what's happening in the present.” Items were rated on a six-point Likert scale from 1 (almost always) to 6 (almost never). Higher scores reflect greater levels of dispositional mindfulness. It has shown good validity and reliability (MacKillop and Anderson, 2007). The Cronbach's α in the current study was 0.83.

#### 2.2.2 Adult ADHD self-report scale symptom checklist (ASRS-v1.1)

The ASRS-v1.1 symptom checklist comprises 18 items, with nine items measuring inattention and nine items measuring hyperactivity/impulsiveness (Kessler et al., 2005). A sample item is “How often do you have trouble keeping your attention on what you are doing?” Each item was scored in the range of 0 (never) to 4 (very often), and all items were summed to generate the total score, ranging from 0 to 72. Higher scores indicate more severe ADHD symptoms. It has good validity in assessing ADHD symptoms in adults (Adler et al., 2019), and it has high test–retest reliability even in adults without ADHD (Silverstein et al., 2018). The Cronbach's α in the current study was 0.89.

#### 2.2.3 Three-stimulus novelty oddball task

**Figure 1:**
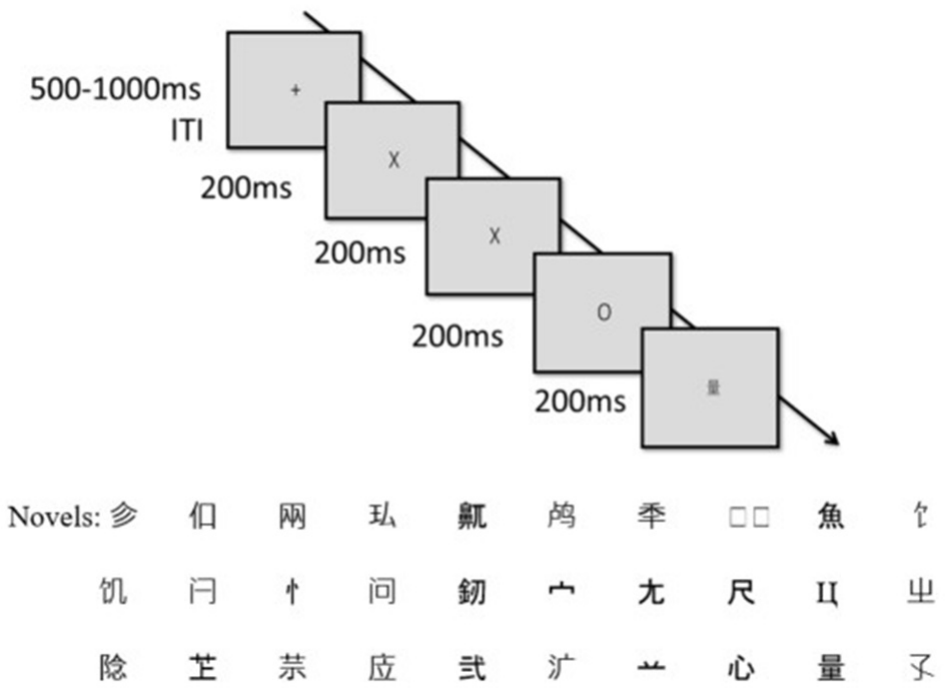
A sample trial in the 3-stimulus visual oddball task and the novels.

The 3-stimulus novelty oddball task consisted of three types of stimuli: 70% standard letters (“X”), 15% target letters (“O”), and 15% novel letters (45 unique Chinese characters; Figure 1). There were a total of 200 trials, evenly divided into two blocks. A trial began with a fixation mark (‘+'), which was randomly jittered between 500 and 1,000 ms to avoid subject expectancy effects on stimulus presentation time, followed by the presentation of one of the three stimuli for 200 ms. Targets did not appear consecutively. Participants were instructed to press key “1” when they saw the target letter O. Novel stimuli engage automatic orienting attention because each novel stimulus is unique, but not endogenous attention because it is task-irrelevant (Katayama and Polich, 1996; Squires et al., 1975); whereas the targets engage endogenous attention because they are rare and task-relevant. The standards do not engage either type of attention because they are neither novel nor task-relevant, thus providing the contrast condition to the novels and targets. Prior to the experimental trials, participants performed 10 practice trials with no EEG recorded. The stimuli were presented in black on a gray background and were presented using E-Prime (Psychology Software Tools, Inc., Pittsburgh, PA).

#### 2.2.4 Auditory device

The auditory tracks from the two interventions were presented through PreSonus Audiobox USB (PreSonus^®^ Audio Electronics, Inc., Baton Rouge, Louisiana). Participants were fitted for foam eartips from E-A-R Link (a 3M multinational conglomerate company, Saint Paul, MN) through insert tube headphones. Both audio files were played at ~75 dB.

#### 2.2.5 Blood pressure monitor device

The ReliOn BP100 Upper Arm Digital Blood Pressure Monitor gave three readings: systolic blood pressure (SBP), diastolic blood pressure (DBP), and heart rate/min. Participants were informed if their blood pressure or heart rate was outside the normal range (< 120 mmHg systolic; < 80 mmHg diastolic; 60–100 beats/min).

#### 2.2.6 EEG acquisition and preprocessing

**Figure 2:**
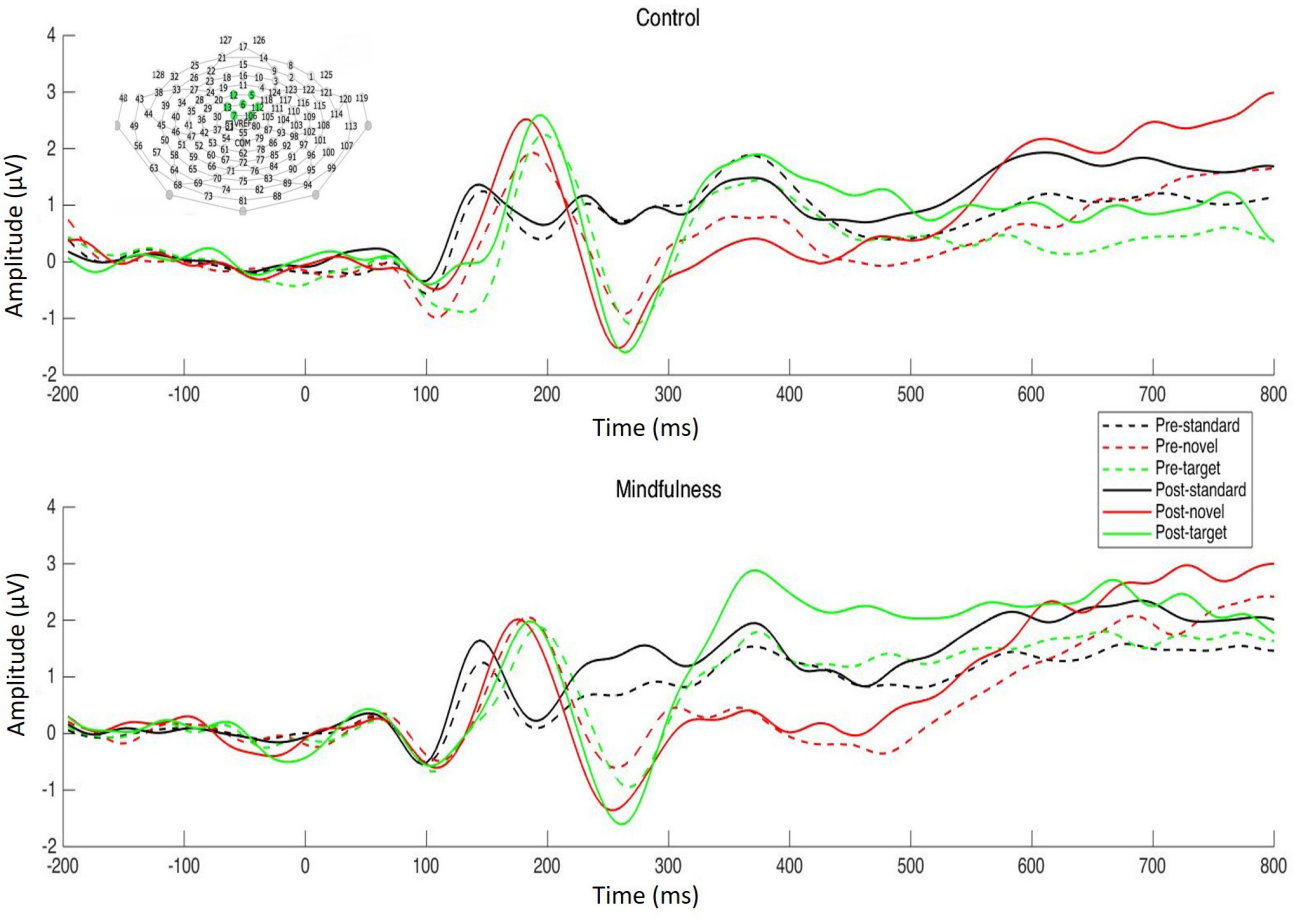
Pre- and post-intervention ERP waveforms to the 3-stimulus visual oddball task averaged over the frontal electrode site (top: control group; bottom: mindfulness group).

**Figure 3:**
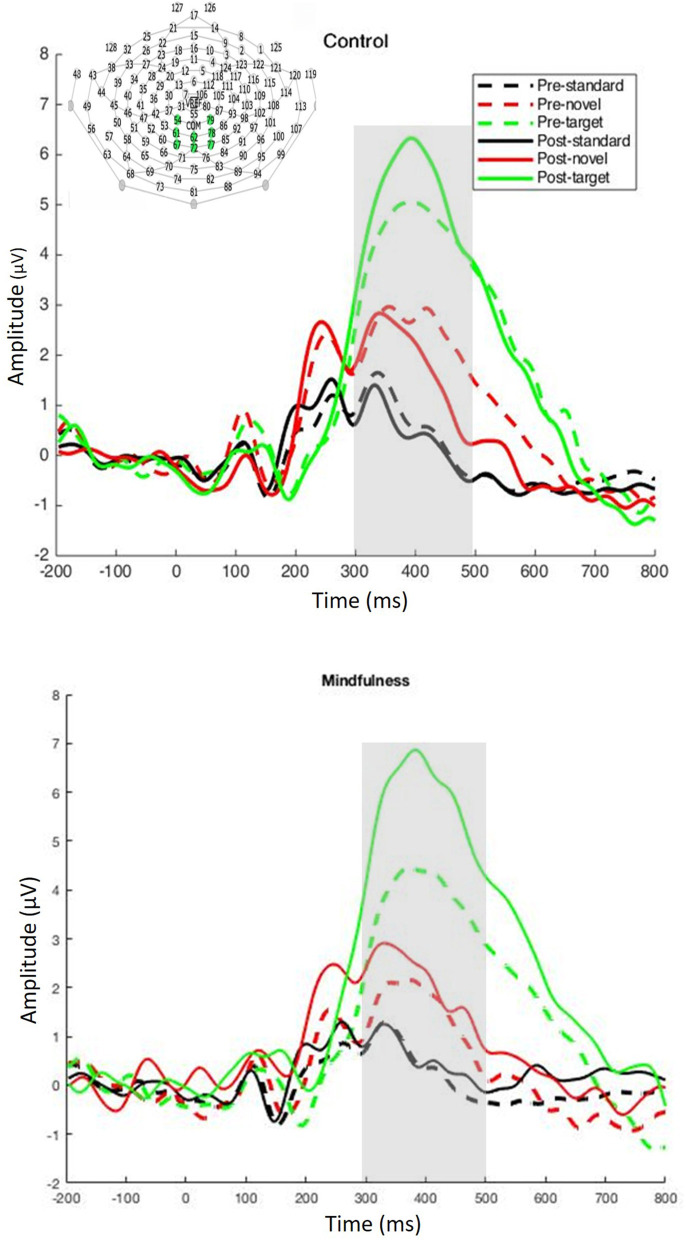
Pre- and post-intervention ERP waveforms to the 3-stimulus visual oddball task averaged over the parietal electrode site. The analysis window is highlighted in gray (top: control group; bottom: mindfulness group).

Electroencephalographic data were acquired using a 128-electrode Geodesic Sensor Net (Electrical Geodesics, Inc./Magstim EGI, Eugene, OR, USA), sampled at 250 Hz, and referenced to the vertex with analog filtering between 0.1 and 100 Hz. The EEG was low-pass filtered at 20 Hz and segmented into 1,000-ms epochs with 200 ms before and 800 ms after stimulus onset. Epochs were then screened for artifacts (e.g., eye blinks and movement, muscle noise) and faulty channels using a combination of visual inspection and the EGI Netstation 5.4.0 artifact detection tool with default settings. Artifact-free epochs were sorted by stimulus and averaged to create the ERPs. Averaged ERPs were then baseline corrected over the 200-ms pre-stimulus period and re-referenced to the average reference. The resulting individual ERPs were averaged across participants within each group to create the grand-averaged ERPs. Grand average ERPs are displayed over the frontal electrode site for P3a and the parietal electrode site for P3b (Figures 2, 3). The ROI for P3a included electrodes 5, 6, 7, 12, 13, 106, 112, and for P3b included electrodes 54, 61, 62, 67, 72, 77, 78, and 79. Average trial numbers are displayed in Table 1. P3a amplitude was defined as the mean amplitude from the time window ranging from 200 to 300 ms post stimulus onset at electrode site FCz. P3b amplitude was defined as the mean amplitude in a time window ranging from 300 to 500 ms post stimulus onset at electrode site Pz.

**Table 1:** Mean number of trials per condition in the novelty oddball task.

**Group**	**Time**	**Standard**	**Target**	**Novels**
Mindfulness	Pre-intervention	96	24	20
	Post-intervention	87	21	18
Control	Pre-intervention	104	25	22
	Post-intervention	94	23	20

### 2.3 Interventions

#### 2.3.1 Mindfulness intervention

Consistent with the two-component proposal of mindfulness practice, we employed a specific and simplified form of mindfulness—mindful breathing, which is central to all forms of mindfulness practice and suitable for meditation-naïve individuals (Bing-Canar and Compton, 2015; Norris et al., 2018). A 10-min guided mindful breathing audio recording (Adams et al., 2013; Vinci et al., 2014) was developed based on the excerpts of Kabat-Zinn (1994). The instructions specifically asked the participants to note their breath and other sensations that may be occurring while doing this with an attitude of nonjudgment and acceptance (see Supplementary material). The protocol was based on findings indicating that induction periods of 10 min or less were sufficient for producing noticeable changes in subsequent measures of attention (Norris et al., 2018). To enhance engagement, participants were informed that they would complete a survey following the practice. The 6-item Practice Quality-Mindfulness (PQ-M; Del Re et al., 2013) was used as a manipulation check. For each given item statement, participants choose a percentage (0–100%) to indicate the extent to which their experience during mindfulness practice reflected the statement. A sample item is, “During the activity, I attempted to return to my present-moment experience, whether unpleasant, pleasant, or neutral.” Items were summed to obtain a total score.

#### 2.3.2 Control intervention

A 10-min audio recording about zero-waste living was retrieved from Youtube.com (Singer, 2015; see Supplementary material). The control audio was chosen to avoid overlapping components with mindfulness, such as focusing on personal experience and maintaining an attitude of non-judgment and acceptance. To enhance engagement, participants were informed that they would complete a survey following the recording: which of the following is not mentioned as a benefit of zero-waster living? A. Eat better; B. Save money; C. Lose weight; D. Need less sleep.

### 2.4 Procedure

Participants completed two experimental tasks as part of a larger research protocol. The current paper focuses on data from the oddball task. Results from the additional tasks will be presented in separate manuscripts to maintain clarity and focus. Consented participants first completed the questionnaires (demographic questionnaires, MAAS, and ASRS-v1.1) on a computer in the testing room and then took a baseline blood pressure measurement (BP1). Participants were then fitted with an EEG net and completed the 3-stimulus oddball task in a sound-attenuated and dimly lit room in the Department of Psychology lab. Following the initial oddball task, participants were fitted with foam eartips for the intervention session. The intervention was administered once to each participant in an individual session. Participants were assigned to groups using a predetermined alternating sequence (e.g., control, experimental, control, etc.) based on their arrival order. While not truly random, this quasi-random assignment approach helped ensure balanced group sizes. The mindfulness group listened to a 10-min mindfulness audio, and the control group listened to a 10-min TED talk about green living. Immediately following the intervention, a post-intervention blood pressure (BP2) was measured, and participants completed the post-intervention 3-stimulus oddball task. During the 1-min inter-block break in the 3-stimulus oddball task, the mindfulness group was instructed to continue practicing mindfulness as guided during the intervention, while the control group was simply instructed to rest. A post-intervention blood pressure (BP3) was measured following the 3-stimulus oddball task. Each blood pressure measurement was averaged from two readings taken with a 1-min interval. Finally, the mindfulness group completed the survey, and the control group completed the manipulation check survey. Participants were debriefed on the study's aims and granted course credit for their participation.

### 2.5 Statistical analysis

Statistical analysis of the data was conducted using SPSS version 26.0. Group differences in demographic data were analyzed using a chi-square test; group differences in age, MAAS score, and ASRS score were analyzed using independent *t*-tests. Blood pressure and heart rate were analyzed using separate 2 (Group: mindfulness, control) by 3 (Time: time 1, time 2, time 3) mixed ANOVAs, with Group as the between-subjects variable and Time as the within-subjects variable. We derived mean ERP amplitude measures for each participant per group. The behavioral and ERP analyses were submitted to a 2 (Group: mindfulness, control) by 2 (Time: pre-intervention, post-intervention) by 3 (stimulus: frequent, rare, novel) mixed analysis of variance (ANOVA), with Group (mindfulness, control) as the between-subjects variable and time (pre-intervention, post-intervention) and stimulus (frequent, rare, novel) as the within-subjects variables. The Bonferroni correction was applied to correct for multiple comparisons. Bayes factor (BF) *t*-test (Rouder et al., 2009) was conducted to examine non-significant ANOVA results. If the initial repeated-measures ANOVA indicated significant improvements in outcome measures from pre-test to post-test across both groups, we would conduct a Bayesian independent samples *t*-test to compare the extent of improvement between the Mindfulness group and Control group. Moderation analyses were conducted using the SPSS PROCESS package (Hayes, 2018), which included the ASRS total score and subscale scores as moderators to examine the relationship between group and change in attention, indexed by the difference in P3b to target using post minus pre (ΔP3b), as moderated by ADHD symptoms.

## 3 Results

### 3.1 Participant characteristics

Demographic information is summarized in Table 2. There were no pre-existing group differences in age, gender, or race/ethnicity. The MAAS score did not differ between the mindfulness group (*M* = 3.85, *SD* = 0.78) and the control group (*M* = 3.80, *SD* = 0.77), *t*_(1, 119)_ = 0.36, *p* > 0.05. There was no difference in the ASRS score between the mindfulness group (*M* = 30.92, *SD* = 9.79) and the control group (*M* = 30.35, *SD* = 9.94), *t*_(1, 119)_ = 0.32, *p* > 0.05, nor for ASRS-inattention, *t*_(1, 119)_ = 0.11, *p* > 0.05 or ASRS-impulsivity, *t*_(1, 119)_ = −0.49, *p* > 0.05. Among the current sample, 54 participants (nearly 50%) fell in the 17–23 range on inattention and 29 for impulsivity; there were 13 individuals who scored above 24 on inattention but only 6 on impulsivity.

**Table 2:** Demographic information (*N* = 121).

**Item**	**Category**	**Control group (*n* = 59)**	**Mindfulness group (*n* = 62)**	**χ^2^ or t**	***p***
Gender	Men	28%	35%	1.34	0.25
	Women	72%	65%		
Race	White	70%	65.5%	7.18	0.30
	Asian	17.23%	16.7%		
	Black or African American	3.5%	6.10%		
	Mixed/Multiracial	7.7%	10.7%		
	Native American	1.5%	0		
	Other/Not Specified	0	1.52%		
Age	18–21	73.8%	81.8%	0.86	0.39
	22–31	26.2%	18.2%		

### 3.2 Outcomes

#### 3.2.1 Physiological data

There was a main effect of Time for systolic blood pressure, *F*_(2, 206)_ = 11.24, *p* < 0.001, $\eta_{\text{p}}^{2}$ = 0.098, diastolic blood pressure, *F*_(2, 206)_ = 7.61, *p* < 0.01, $\eta_{\text{p}}^{2}$ = 0.07, and heart rate, *F*_(2, 206)_ = 41.4, *p* < 0.001, $\eta_{\text{p}}^{2}$ = 0.29. Follow-up pairwise comparisons revealed that all three measures were higher at pre-intervention than post-intervention, *p* < 0.01, and both systolic blood pressure and heart rate at post-intervention did not differ from that measured at end-of-task, *p* > 0.05, while diastolic blood pressure was higher at end-of-task than at post-intervention, *p* < 0.001. There was no group effect or other interaction effects.

#### 3.2.2 Behavioral data

Two (Group: mindfulness, control) by 2 (Time: pre-intervention, post-intervention) by 3 (Stimulus: frequent, rare, novel) mixed ANOVAs on reaction time and error rate revealed no significant main effects or interactions involving Time or Group (Table 3).

**Table 3:** Descriptive statistics of reaction times and error rate in the 3-stimulus oddball task.

**Group**	**Time**	**Reaction time**	**Error rate**
Mindfulness	Pre-intervention	360.59 (48.02)	0.006 (0.01)
	Post-intervention	357.81 (44.07)	0.003 (0.01)
Control	Pre-intervention	375.30 (42.83)	0.004 (0.01)
	Post-intervention	371.27 (45.10)	0.003 (0.01)

#### 3.2.3 ERP data

**Figure 4:**
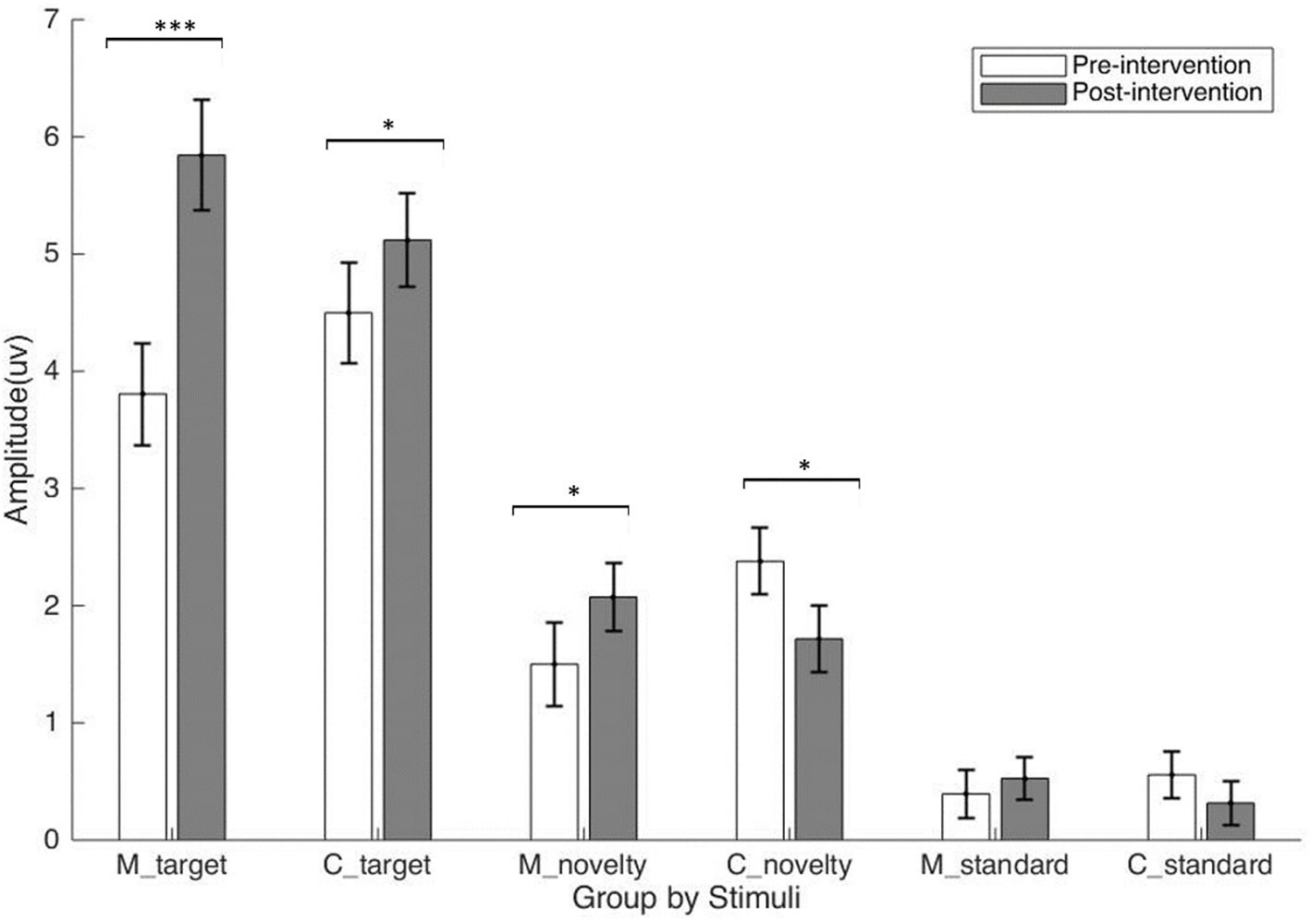
Mean P3b amplitude for group × time × stimuli interactions (mindfulness: *n* = 56; control: *n* = 45). The error bar represents the standard error of the mean. * *p* < 0.05; ***p < 0.001.

Visual inspection of the ERPs did not reveal a P3a; therefore, no follow-up analysis was performed (Figure 2). A main effect of stimuli was observed, *F*_(2, 198)_ = 181.88, *p* < 0.001, $\eta_{\text{p}}^{2}$ = 0.65. P3b elicited by targets (*M* = 4.82, *SE* = 0.30) was larger than P3b elicited by standards (*M* = 0.45, *SE* = 0.13) and novels (*M* = 1.92, *SE* = 0.19); and a main effect of time was observed, *F*_(1, 99)_ = 11.07, *p* < 0.01, $\eta_{\text{p}}^{2}$ = 0.10. P3b was larger at post-intervention (*M* = 2.60, *SE* = 0.17) than pre-intervention (*M* = 2.19, *SE* = 0.19). There was a Stimulus × Time interaction effect, *F*_(2, 198)_ = 24.88, *p* < 0.001, $\eta_{\text{p}}^{2}$ = 0.20. Simple effect analysis revealed that P3b was larger at post-intervention than pre-intervention for targets, *p* < 0.001, but not for standards or novels. There was also a Time × Stimuli × Group interaction, *F*_(2, 198)_ = 3.06, *p* < 0.05, $\eta_{\text{p}}^{2}$ = 0.03. P3b to targets increased in both the mindfulness group, *p* < 0.001, and the control group, *p* < 0.05. P3b to novels also increased in the mindfulness group, *p* < 0.05, but decreased in the control group, *p* < 0.05. Grand average ERPs are plotted in Figure 3. Mean P3b amplitude for Group × Time × Stimuli interactions is displayed in Figure 4. The BF *t*-test on the difference score of P3b to target revealed strong evidence that the increase in P3b to target in the mindfulness group is larger than that in the control group, *BF*_10_ = 81.87.

#### 3.2.4 Moderation by ADHD symptoms

**Figure 5:**
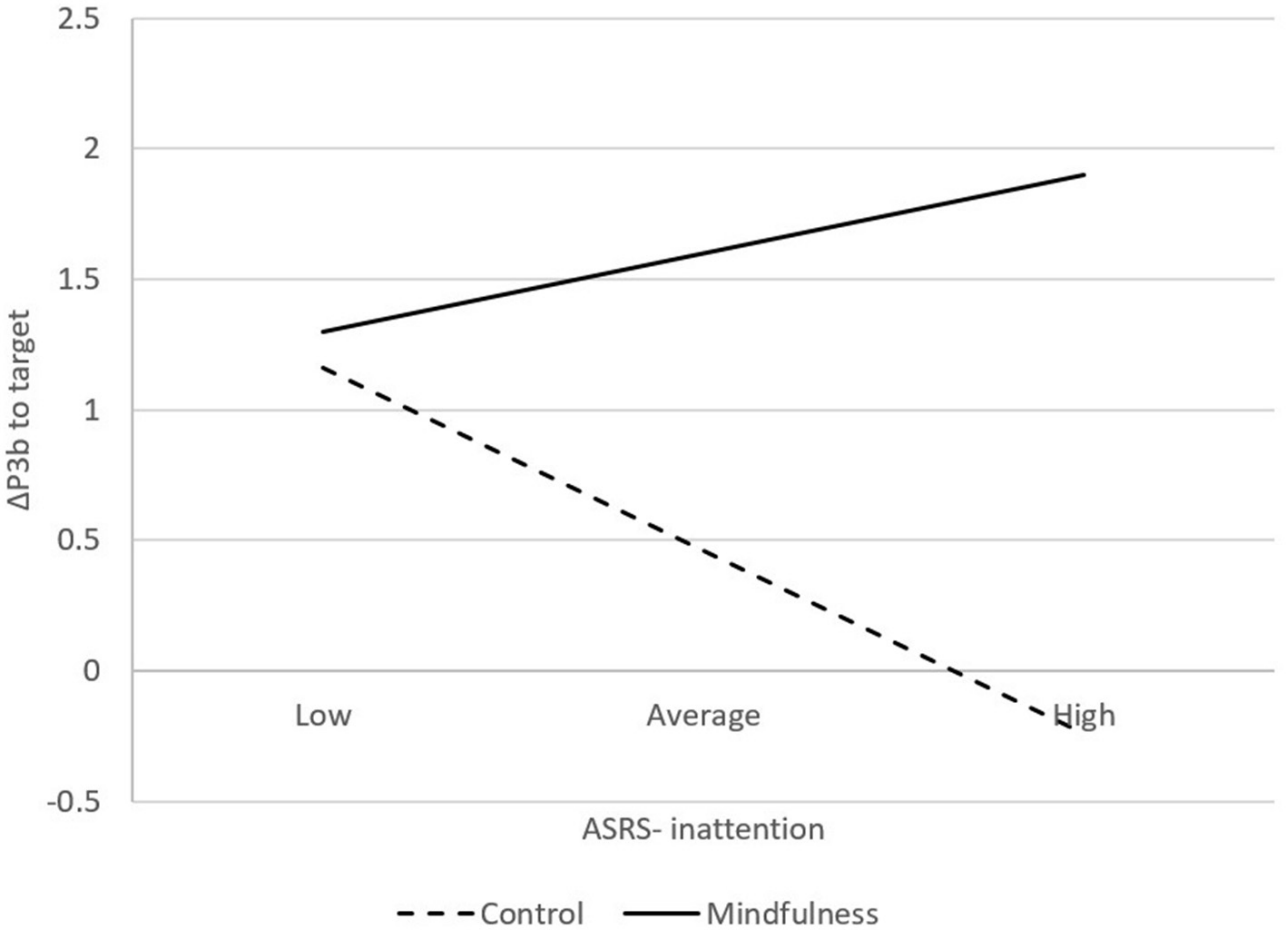
Inattention symptom moderated the relationship between group and ΔP3b to target (mindfulness: *n* = 56; control: *n* = 45).

The ASRS score was normally distributed. Moderation analysis revealed a significant Group by Inattention interaction, *F*_(3, 97)_ = 4.38, *p* < 0.05, suggesting that as inattention score increases, P3b to target from pre- to post-intervention tended to increase in the mindfulness group but decrease in the control group (Figure 5). Specifically, a significant effect emerges when the inattention score is above 15.81. There was no significant interaction effect for the total ADHD score or the impulsivity score.

## 4 Discussion

To our knowledge, this is the first study to investigate the moderating effect of baseline attention capacity on the attention-enhancing benefits of mindfulness practice. In a pre-post between-groups design, the present study found that both the mindfulness and control interventions altered autonomic physiology, as evidenced by decreased systolic blood pressure, diastolic blood pressure, and heart rate. Although both the 10-min mindfulness of breathing exercise and listening to a talk improved attentional resources, as indexed by a larger P3b to targets from pre- to post-intervention, the improvement in attention was greater in the Mindfulness group than in the Control group, as evident in the Bayesian analysis. Inattention, but not impulsivity, moderated the relationship between mindfulness practice and attention. Specifically, as inattention symptoms increased, P3b to the target tended to increase from pre- to post-intervention in the mindfulness group but decrease in the control group.

Inattention moderated the relationship between mindfulness practice and attention among this sample of meditation-naïve college students. In particular, the difference in change in P3b to the target tended to emerge when the inattention score was above 15.81. A previous study suggested that a sum score above 17 on either subscale is classified as likely to have ADHD (Yeh et al., 2008). As inattention symptoms increased, P3b to the target tended to increase from pre- to post-intervention in the mindfulness group but decreased in the control group. This finding is consistent with a review that suggests the efficacy of mindfulness interventions in reducing ADHD symptoms is moderated by at-risk status, meaning that the efficacy is more robust among individuals at risk for inattentiveness and impulsivity (Vekety et al., 2021). The moderating effect was not found for impulsivity because the impulsivity symptom is less severe than the inattention symptom. Yeh et al. (2008) suggested that individuals with a sum score of 17–23 on either subscale are classified as likely to have ADHD, and a sum score of 24 or greater on either subscale is highly likely to have ADHD (Yeh et al., 2008). Nearly half of the current sample (*n* = 54) fell within the 17–23 range on inattention and 29 on impulsivity; 13 individuals scored above 24 on inattention, but only 6 scored above 24 on impulsivity. Together with the results from Bayesian analysis, these findings suggested that mindfulness intervention was superior to listening to a talk about green living in improving attention when individuals were at risk of ADHD.

The results partially supported the main hypothesis that a 10-min mindfulness of breathing exercise improved attention as indexed by increased P3b amplitudes to targets from pre- to post-intervention. This finding is consistent with prior research demonstrating that mindfulness practices can enhance attentional processes (see Chiesa et al., 2011, for a review). The enhancement of attention assessed by P3b has been shown to be a result of both brief mindfulness intervention, e.g., a one-session 6-min guided mindfulness intervention (Lakey et al., 2011), and long-term mindfulness meditation, including mindfulness meditators with an average of 13.1 years of experience (Jo et al., 2016) and vipassana meditators with an average experience of 7.5 years (Delgado-pastor et al., 2013). While the current findings are not novel, they contribute to the growing body of evidence supporting the attentional benefits of short-term mindfulness interventions, particularly as measured by neurophysiological markers such as the P3b.

Surprisingly, the controls also showed a larger post-intervention P3b to targets, indicating that attentional resources also benefited from listening to a 10-min TED talk about green living. One possible explanation is that relaxation provides restorative opportunities for attention (Rutschman and College, 2004; Yesavage and Jacob, 1984). Participants in the control group were instructed to rest during the break between task blocks, and they showed reduced BP, suggesting increased relaxation. Notably, previous between-group studies that also compared mindfulness intervention to a relaxation condition did not report a relaxation effect on attention (Lakey et al., 2011; Mrazek et al., 2012). The reason for the discrepant findings might be that previous studies only measured attention after, not before, the intervention, which only allows for the examination of the group effect, rather than the change in attention in response to the intervention.

Although both interventions improved endogenous attention, the mindfulness intervention was superior to the control group, as indicated by the results from Bayesian analysis. While the improvement in attention in both groups may have benefited from increased relaxation, the mindfulness group might also have benefited from attention regulation, which is more effective in improving attention than pure relaxation (Bishop et al., 2004). Alternatively, mindfulness might be more relaxing than the control condition. Future studies could include measures to assess such constructs as self-reported relaxation and attention regulation to further understand their impact.

The increased P3b to target resulted from the interventions rather than a practice effect or time passing, because the P3b to target has good test-retest reliability in the visual modality (Cassidy et al., 2012; Ip et al., 2018; Jodo and Inoue, 1990). For example, studies have observed stable P3b to target across six blocks with a 20-min inter-block interval (Geisler and Polich, 1994) and across two blocks with a 1-h interval (Wesensten et al., 1990). These inter-block intervals resemble the time intervals between the novel oddball tasks in the current study. To the contrary, practice effects may even reduce the P3b, which has been found to habituate when the inter-block interval is relatively short, e.g., across 10 blocks with a 10-min inter-block interval (Ravden and Polich, 1998) and across three blocks with a 30-s inter-block interval (Wesensten et al., 1990). Short inter-block intervals are thought to familiarize participants with the task and reduce the attention required to perform it (Kramer et al., 1986), especially when the task is easy (Kok, 1990; Strayer and Kramer, 1990; Kok, 1997). To the best of our knowledge, no reports of increased P3b to the target have been documented through repeated testing or with the passage of time.

In the current study, both targets and novels produced a P3b. Relative to task-irrelevant novel stimuli, task-relevant targets contributed substantially more to the P3b. Unlike P3b to target, the cognitive correlates of P3b elicited by novels largely remain unclear (Friedman et al., 2001; Polich, 2003, 2007). Some argue that P3b to novels indexes task-relevance processing of the novels, with larger P3b indicating greater task relevance (Gaeta et al., 2003). Others propose that P3b to novels reflects a classification process, with larger P3b indicating greater processing due to the unfamiliarity of the novels (Friedman et al., 2001). This could be due to the characteristic of mindfulness practice being “attentive to and aware of what is taking place in the present” (Brown and Ryan, 2003, p. 822), even for task-irrelevant stimuli.

Novels did not elicit a P3a in the present novelty oddball task. The absence of the P3a might be because the task is too easy to elicit a P3a (Comerchero and Polich, 1998; Hagen et al., 2006). Difficult tasks may engage the processing of novels more strongly by allocating more attention resources to the stimuli than easier tasks (Hagen et al., 2006). Moreover, previous visual 3-stimulus oddball tasks that have successfully elicited a P3a have made the novels perceptually salient (e.g., complex in form or larger in size than the standards and targets), such as colorful abstract-type drawings (Courchesne et al., 1975) or a large black/white checkerboard square as novels while targets and standards are blue circles (Conroy and Polich, 2007). The deviance of the novels from the standards and targets might be too small to elicit a P3a in the current 3-stimulus oddball task, as the novels were in the same color (i.e., black) and size as the standards and targets.

Several limitations should be noted. First, we only measured trait mindfulness, not state mindfulness; therefore, we were unable to examine the change in state mindfulness resulting from the intervention. It should be noted that no differences emerged between groups in trait mindfulness, as measured by the MAAS. Moreover, the generalizability of the improved attention in individuals with elevated inattention symptoms is limited by the current sample, who were not clinically diagnosed with ADHD. To what extent these findings can be transferred to a population with clinically diagnosed ADHD requires further investigation. Finally, we did not explain “attitude of non-judgment and acceptance” to the participants before the intervention. Although the mindfulness intervention audio introduced the concepts of acceptance and non-judgment, prior research suggests that these components often take longer to develop than more immediately accessible aspects of mindfulness, such as attentional focus on the breath (Baer, 2003; Shapiro et al., 2006). Given the central role of acceptance in mindfulness-based interventions, future studies may benefit from explicitly introducing and contextualizing this concept for novices in advance, which could enhance engagement and improve intervention outcomes.

## 5 Conclusion

These findings provide novel insights into individual differences in response to mindfulness training, highlighting the moderating role of baseline attention capacity. That is, brief mindfulness practice can enhance attentional processing, particularly among individuals with higher levels of inattention. While both mindfulness and control conditions yielded physiological benefits and improved attentional neural markers, mindfulness training led to a greater enhancement in attentional allocation as indexed by a significant increase in the P3b amplitude. Notably, the differential impact of mindfulness on attention as a function of inattention symptoms suggests that individuals with attentional difficulties may derive greater cognitive benefits from even brief mindfulness interventions. These results highlight the potential of tailoring mindfulness-based approaches to individual attentional profiles, particularly in educational settings where students often struggle with focus and cognitive overload. For instance, integrating brief mindfulness exercises into classroom routines or academic support programs could offer a low-cost, scalable intervention to enhance students' attentional control, particularly for those with elevated symptoms of inattention. By identifying individuals who are more likely to benefit, educators and mental health professionals could more effectively target mindfulness-based strategies to those who need them most, such as those reporting difficulties with sustained attention. This personalized approach may help improve academic performance, classroom engagement, and overall wellbeing among college students navigating high cognitive demands. Moreover, when applying mindfulness-based interventions to other clinical conditions, it is essential to consider individual differences in attention, as these can significantly impact treatment outcomes. The current study is among the few that highlight how variability in attentional capacity can moderate the effects of mindfulness training. Future research should further explore how other individual traits, particularly those related to cognitive functioning, shape responsiveness to mindfulness.

## Data Availability

The raw data supporting the conclusions of this article will be made available by the authors, without undue reservation.
