# Supplementary material for: A brief mindfulness intervention improves electrophysiological markers of attention in meditation-naïve individuals: the moderating role of inattention symptoms
Source: Front Psychol. 2025 Aug 21;16:1629282. doi: 10.3389/fpsyg.2025.1629282 (PMC12408574; doi:10.3389/fpsyg.2025.1629282)
Supplement: Supplementary file 1 [file Supplementary_file_1.docx]

Supplementary. Transcript for Interventions

# Mindfulness intervention

***(Start: 00:01)*** This is the sitting meditation on the breath. While sitting down in your chair, begin by placing your feet flat on the floor. Try to sit up straight and relax your shoulders, your neck, and place your hands in your lap or on your knees. Now as you settle into a comfortable position, commit yourself to simply being fully awake, fully present for these next few moments. And if you feel comfortable with it, gently close your eyes. Otherwise, just look down toward the floor.

***(00:43)*** *6 second silent period*

***(00:49)*** Begin by bringing your attention to your breath. Noticing the breath as it moves in and out of your body.

***(00:58)*** *5 second silent period*

***(01:03)*** Perhaps focusing on the sensation of the breath that moves in and out of your nose. Noticing the air moving here and any temperatures that come with the in breath and the out breath.

***(01:16)*** *5 second silent period*

***(01:21)*** Or perhaps pay attention to the rise and fall of the chest and abdomen as you breathe in and out. Just allow you attention to fall wherever it naturally does when you notice the breath.

***(01:35)*** *21 second silent period*

***(01:56)*** And naturally your mind may wander off to something else during this exercise. You may start to listen to sounds that you are hearing or on some other type of physical sensation. And whenever this happens just bringing your attention back to the breath, back to your breathing.

***(02:17)*** *24 second silent period*

***(02:41)*** You may notice thoughts come up about something that you’re planning to do in the future or even something that’s already happened earlier today. Just notice what’s on your mind and how your body is feeling when this happens. Acknowledging that these thoughts have come up, whatever they are, without judging or evaluating them. And then just gently let them go. Bringing your attention back to the breath. Focusing on the sensation of the breath moving in and out of the body. Again, wherever you notice that the most.

***(03:17)*** *32 second silent period*

***(03:49)*** Noticing where the mind is right now. And bringing your attention back to the breath if it has gone elsewhere. Just breathing in and breathing out.

***(04:01)*** *36 second silent period*

***(04:37)*** And each time that you notice that your mind has gone someplace else, wherever that may be, just bring your attention back to the breath. And if the mind wanders of a thousand times, you simply bring it back a thousand times. Intentionally cultivating an attitude of patience and gentleness towards yourself. This means choosing, as best as you can, to not react to or judge any of your thoughts, or feelings, or perceptions. Instead, just reminding yourself that absolutely anything that comes into the field of awareness is okay. Noticing when this happens. Bringing attention to it. And then slowly bringing your attention and focus back to the breath.

***(05:32)*** *30 second silent period*

***(06:02)*** We simply sit and breathe and observe it. Staying open and awake in the present moment right here, right now. And this may be a continual process of seeing and letting be, seeing and letting go. Rejecting nothing. Pursuing nothing. Just dwelling in the stillness and calmness as the breath moves in and out.

***(06:30)*** *43 second silent period*

***(07:13)*** Where is the mind right now?

***(07:15)*** *34 second silent period*

***(07:49)*** If you’d like, commit yourself to bringing this attitude of attention and acceptance with you throughout your day. Being fully aware of the present moment. Noticing any thoughts or feeling that may arise without judging them. Just being right here, right now. Accepting the present moment and accepting yourself no matter what happens. And remember that you can always bring your focus back to your breath, back to the sensations of the present moment, to help cultivate the sense of attention and acceptance.

***(08:25)*** *11 second silent period*

***(08:36)*** Continuing to focus on the breath. Just noticing the breath. Breathing in and breathing out. Breathing in and breathing out.

***(08:52)*** *40 second silent period*

***(09:32)*** Go ahead and take just a few more breathes. Being fully present and fully aware in this moment.

***(09:40)*** *9 second silent period*

***(09:49)*** And then, whenever you’re ready, go ahead and open your eyes.

***(End: 09:54)***

# Green living

I live a zero waste lifestyle, and I have for the past three years. Now, zero waste, that's a pretty big idea. Right? So let me define it for you. To me living zero waste means that I don't make any trash. So no sending anything to landfill, no sending anything in a garbage can, and no spitting gum on the ground, and walking away. Right? No trash. This is a big concept, and this all started when I was an environmental study student at NYU. My senior year, I was taking a course called: “The Environmental Studies Capstone course”, which is essentially the culminating course that all environmental study students need to take in order to go out into the world, and make it a more sustainable place. Well, there was a girl in this class, and every class she would have this big plastic bag, with a plastic clamshell full of food, a plastic fork and knife, a plastic water bottle, and a plastic bag a chips, and she would eat all of this, and then class after class, would just throw it in the trash. This was really frustrating, because here we were these environmental study students trying to make the world a better place, and there she was, throwing all this stuff into the garbage. One day after class, feeling still particularly upset about watching her throw everything away, I went home to make dinner, and I opened my fridge, and noticed something that I had never seen before. Every single thing in my fridge was in one way or another packaged in plastic, and I couldn't believe it. You know I was getting so mad at this girl for making so much plastic trash, and it turns out that I was just as bad. I was that girl, and so I made a decision in that moment. I was going to stop using plastic. Well, quitting plastic, not so easy of a thing. Right? When you think about your everyday life, you wake up in the morning, you go into the bathroom, and you brush your teeth. What is your toothbrush made out of? Plastic. What is your toothpaste probably packaged in? Plastic. Your face wash, your moisturizer, your contact solution. So many things that are in our everyday lives come packaged in plastic, and so I realized that if I was going to move away from plastic, the only way that I was going to do that was to learn how to make my products myself. Well, I don't know about you, but I certainly didn't know how to make deodorant. I didn't have the recipe just hanging out in my back pocket, and so I realized that I had to do some research, and while I was doing research online, I came across a blog called the "Zero Waste Home" started by a woman named Bea Johnson who is a wife, and mother of two kids, out in Mill Valley, California, and the four of them live a completely zero waste life. When I learned about Bea, and her family, my mind was completely blown. I thought that I was doing the best thing for the planet by not using any plastic. But the idea that I didn't have to produce any trash, was so empowering, and so inspiring, and it made perfect sense. Right? Because I was this Environmental Studies student, I cared about the environment, studied sustainability, talked about sustainability, protested for sustainability. But I realized, that I wasn't actually implementing any of those values into my day-to-day life, and so I made the decision to go zero waste. So let me break it down for you, and tell you some of the things that I did in order to make this transition a little easier. The first thing that I did was I stopped buying packaged food. So instead of going to the store, and buying things packaged in paper, and glass, and plastic, I started bringing my own jars, and bags to the store to fill with bulk, or package-free items. I also started buying my fruit, and vegetables from the farmer's market. So, package-free. The second thing that I started doing was I started making all of my own products. Before I started living this lifestyle, my boyfriend at the time, used to brush his teeth using baking soda, and I thought he was probably the grossest person in the entire world. Right? There’s no way that you can possibly get your teeth clean using something like baking soda, it's gross. Well fast-forward, and it turns out that the first product that I made was toothpaste, made with baking soda. So overtime I started making all of my own products. When I would run out of something, instead of going to the store, and buying a new one, I would learn how to make it myself. So when I would run out of lotion, I learned how to make it myself. Run out of deodorant, learn how to make it myself. Over time, all of the things I had previously purchased, were now, ones that I made myself. The third thing that I started doing, was shopping second-hand. So instead of buying new clothing, and putting new waste into the waste cycle, I would buy things that were totally recycled, second-hand. So not making any new trash. The fourth thing that I did was I downsized. So I focused on having only the things that were truly necessary, and that I really needed. Well this was really, really hard because I'm the kind of person who's really sentimental, and I can tell you as to why a toothpick needs to be in my life. But after I really got through that process, and I completely downsized, I realized that I had so many fewer things in my life, my home was less cluttered, and everything was easier to clean. And when you have fewer things you realize that you take better care of them. Right? When you take better care of your things you don't have this mentality like: "If I don't want this anymore I'll just throw it out and I’ll get a new thing later. “No, I only had a few things and so I took care of them, and wasn't sending anything to the landfill. All this must sound pretty difficult. Right? I assure you, it's not that hard. I’m just an average, lazy person, and I wouldn't live this lifestyle if it was difficult. In fact the benefits of living this lifestyle far outweigh any of the negatives that you can imagine. The first benefit is that I save money. So I save money when I buy my food, and the products, and when I make my own products, because I'm not paying for the embedded cost of packaging, so things are cheaper. I’m also saving money by shopping completely second-hand, because second-hand clothing is usually less expensive than new clothing. I am also saving money because I've downsized. I don't go shopping all the time now and you know just buy things on impulse. I only have what I really need. The second benefit is that I eat better. When I go shopping now I don't have the option to buy processed food products, package-free, and so now my diet consists of things like fresh fruit and vegetables, or bulk you know, greens, and nuts that I buy with my jars and my bags. And so when you eat better, you feel better. Over these past few years, I've noticed that my weight has stabilized, I have more energy, I need less sleep, and when you're eating better, and you feel better, and you save money, you're happier. But besides those things I'm happier, because for the first time in my life, I’m living in direct alignment with my values. And why is this important? Right? Waste. Well, waste is a really big problem. In fact the average American person produces approximately 4.4 pounds of trash per person per day. Over the course of a year, that's like taking 8.5 of your best friends, and throwing them in the trash. Don't do that, it's not nice. So, if you care about your friends, and you don't want to throw them away, and you think that it's possible for you to reduce how much trash you're producing, I have three simple steps for you.

The first step is to actually look at your trash, and understand what it is. Because you can't solve a problem of having a lot of waste until you know what it is. So when I did this exercise, I realized that I had three main sources of trash. The first was food packaging, and so I learned how to shop in bulk or package-free. The second was product packaging, and so I learned how to make all of my own products. And the third was organic food waste, and so I learned how to compost. And just by identifying those three sources of waste and eliminating them, I have reduced my trash by about 90%. The second thing that I'd like to suggest is picking at the low-hanging fruit. So doing little things, one-time changes in your everyday life that have a large-scale, and long-term positive impact. This includes things like using a reusable bag instead of a plastic or paper bag. Or using a stainless steel, or glass water bottle, instead of buying plastic water bottles. Over the course of, you know, however long, you realize that these little changes actually add up, and make a big difference. The third thing that I'd like to suggest is the DIY or actually learning how to make your products yourself. Now I absolutely love doing this because when you go to a store, and you have to buy products you kind of have to settle, and accept them as they are. Right? If you don't like the way they smell, too bad. If you don't like the way they feel, sorry. If you don't like what they're packaged in, you don't have a choice. But for me, since I make all my own products, if I don't like the way they smell, I change the scent. If I don't like the ingredients in them, I change it. If I don't like the packaging, it's my choice. And so by making my own products I have complete control over what I'm putting in my body
